# Time to antibiotics is unrelated to outcome in pediatric patients with fever in neutropenia presenting without severe disease during chemotherapy for cancer

**DOI:** 10.1038/s41598-022-18168-x

**Published:** 2022-08-18

**Authors:** Christa Koenig, Claudia E. Kuehni, Nicole Bodmer, Philipp K. A. Agyeman, Marc Ansari, Jochen Roessler, Nicolas X. von der Weid, Roland A. Ammann

**Affiliations:** 1 Pediatric Hematology/Oncology, Department of Pediatrics, Inselspital, Bern University Hospital, University of Bern, 3010 Bern, Switzerland; 2 Institute of Social and Preventive Medicine, University of Bern, Bern, Switzerland; 3 Pediatric Oncology, Kinderspital Zürich, University of Zürich, Zurich, Switzerland; 4 Department of Pediatrics, Inselspital, Bern University Hospital, University of Bern, Bern, Switzerland; 5 Pediatric Hematology/Oncology, Department of Women, Child and Adolescent, University Hospital of Geneva, Geneva, Switzerland; 6 Department of Pediatrics, Gynecology, and Obstetrics, Cansearch Research Platform of Pediatric Oncology and Hematology, Faculty of Medicine, University of Geneva, Geneva, Switzerland; 7 Division of Pediatric Hematology and Oncology, University Children’s Hospital Basel, University of Basel, Basel, Switzerland; 8 Kinderaerzte KurWerk, Burgdorf, Switzerland

**Keywords:** Health care, Medical research, Oncology, Signs and symptoms

## Abstract

Fever in neutropenia (FN) remains an unavoidable, potentially lethal complication of chemotherapy. Timely administration of empirical broad-spectrum intravenous antibiotics has become standard of care. But the impact of time to antibiotics (TTA), the lag period between recognition of fever or arrival at the hospital to start of antibiotics, remains unclear. Here we aimed to analyze the association between TTA and safety relevant events (SRE) in data from a prospective multicenter study. We analyzed the association between time from recognition of fever to start of antibiotics (TTA) and SRE (death, admission to intensive care unit, severe sepsis and bacteremia) with three-level mixed logistic regression. We adjusted for possible triage bias using a propensity score and stratified the analysis by severity of disease at presentation with FN. We analyzed 266 FN episodes, including 53 (20%) with SRE, reported in 140 of 269 patients recruited from April 2016 to August 2018. TTA (median, 120 min; interquartile range, 49–180 min) was not associated with SRE, with a trend for less SREs in episodes with longer TTA. Analyses applying the propensity score suggested a relevant triage bias. Only in patients with severe disease at presentation there was a trend for an association of longer TTA with more SRE. In conclusion, TTA was unrelated to poor clinical outcome in pediatric patients with FN presenting without severe disease. We saw strong evidence for triage bias which could only be partially adjusted.

## Introduction

Children and adolescents with cancer and chemotherapy induced neutropenia are at increased risk for infections^1^. Despite improved supportive care, fever in neutropenia (FN) remains a persistent and potentially lethal complication of chemotherapy. Timely administration of empirical broad-spectrum intravenous antibiotics, recommended by current FN guidelines^2–4^, has become standard of care, despite the fact that in the majority of patients with FN viral infections can be detected^5^. Time to antibiotics (TTA) refers to the time span between first recognition of fever or arrival at the hospital, and start of empiric intravenous antibiotic administration^6^. Although it seems intuitive that longer TTA affects safety outcomes, evidence is inconsistent and the question of how fast antibiotics should be administered is still unanswered^7^. Currently TTA < 60 min is used as quality of care measure^8^ and several centers have used considerable resources to reduce TTA^9,10^.

Triage bias, e.g. faster treatment of patients presenting in reduced general condition or at higher risk for poor outcome, has been identified as important confounding factor when analyzing TTA^7,11^. Without bias correction, the true effect of TTA on patient important outcomes may be undetectable. Large delays from fever onset to hospital visit (median 12.5 h; interquartile range (IQR) 6–24 h) were found by a Central American study in children with cancer^12^. Most other studies only measured and analyzed TTA from arrival at the hospital to start of antibiotics^11,13^. Therefore, the influence of potentially important delays prior to hospital arrival are not adequately understood, and may have a fundamental impact on clinical outcome.

This analysis aimed to investigate the association between TTA, the time from recognition of fever to start of antibiotics, and safety relevant events (SRE) in children and adolescents undergoing chemotherapy for cancer. We included information on time that evolved before arrival at the hospital and we attempted to adjust for possible triage bias with a propensity score.

## Methods

### Study design and participants

For this paper we analyzed data collected in the Swiss Paediatric Oncology Group (SPOG) 2015 FN Definition study. In this prospective multicenter study, six out of nine pediatric oncology centers in Switzerland consecutively screened and recruited patients from April 2016 to August 2018. The study was registered at ClinicalTrials.gov (NCT02324231) on 24/12/2014 and was approved by local ethics committees before patient recruitment^14^.The study was done in accordance with the Declaration of Helsinki and the Swiss Law. Data were collected and managed using REDCap electronic data capture tools^15^. Inclusion criteria were age ≥ 12 months and < 18 years, diagnosis of any malignancy, treatment with myelosuppressive chemotherapy expected to last ≥ 2 months, or ≥ 1 cycle of myeloablative chemotherapy followed by autologous HSCT, and written informed consent. Patients after allogeneic HSCT were excluded for safety reasons^14^. The primary aim of the study was to determine the safety of a higher (39.0 °C) versus lower (38.5 °C) fever limit using a non-blinded cluster-randomized controlled non-inferiority design. The 39.0 °C fever limit was found to be both safe and efficacious when compared to 38.5 °C^14^. Secondary results about a risk prediction score for FN with SRE during chemotherapy, have been published recently^16^. Here we analyze information on different times of diagnosis and treatment of FN.

### Procedures and management of fever

The study collected data on FN episodes from in- and outpatients. Inpatients were either hospitalized or assessed during hospital visits. Outpatients developed fever outside the hospital, e.g. at home. No conditions were specified for the place of residence during ambulatory treatment. Distance to the respective center therefore varied, with travel times between few minutes to approximately 3 h. There were no Family Houses or comparable institutions next to the hospitals used for patient lodging. Ear temperature was measured by parents and nurses if fever was suspected, and at least twice daily in inpatients. The parents were instructed to call the hospital if a temperature ≥ 38.5 °C was measured or if the patient’s general performance was reduced, and the responsible local pediatric hemato-oncologist was immediately informed. In inpatients, the nurses acted correspondingly^14^. All temperature measurements were done in the ear with infrared tympanic thermometry using a Braun ThermoScan® 7 device (IRT 6520)^17^ throughout the study. Neutropenia was defined as an ANC < 0.5 G/L, or < 1.0 G/L and expected to decline to < 0.5 G/L within 48 h^18,19^. FN was diagnosed during neutropenia at ear temperatures reaching the randomized limit of 38.5 °C or 39.0 °C, or below this limit with at least slightly elevated temperature (≥ 38.0 °C; or ≥ 37.5 °C in patients repeatedly receiving antipyretics), if the responsible physician decided to do so^14^. Diagnosis of FN implied emergency hospitalization and start of empirical intravenous broad-spectrum antibiotics. Coverage of Gram-positive cocci (except methicillin-resistant Staphylococcus aureus, coagulase-negative staphylococci, and enterococci) and Gram-negative bacteria was required, but a specific anti-anaerobic coverage was not^14^.

### Times

Parents and nurses reported the exact time of relevant temperature measurements (first measurement ≥ 38.5 °C and ≥ 39.0 °C). Time of arrival at the hospital and start of antibiotics were extracted from the patients’ hospital chart. For inpatients time of arrival was defined as time of the phone call to the treating pediatric hemato-oncologist.

**Figure 1:**
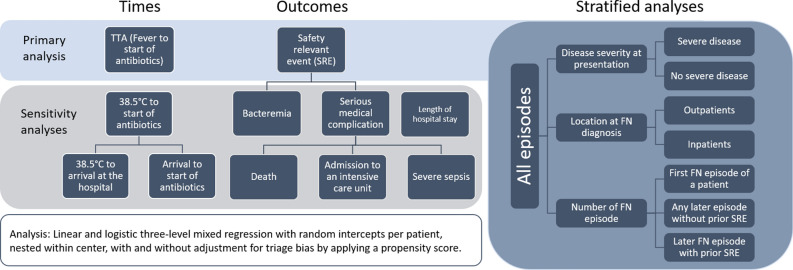
Scheme of analyses: Times, outcomes and stratified analyses.

For this analysis we used different ways to describe time spans between recognition of fever and start of antibiotics (Fig. 1). Primary TTA (furthermore called TTA), was defined as time span from reaching the randomized fever limit (38.5 °C or 39.0 °C) to start of the first dose of antibiotics. We chose this definition of TTA to avoid bias introduced by the randomization of fever limits. Secondary TTAs were the time span from first temperature measurement of 38.5 °C to antibiotics, regardless of the randomized fever limit. This time span was additionally split into time from reaching 38.5 °C to arrival at the hospital and time from arrival at the hospital to start of antibiotics.

### Outcomes

Outcomes were assessed per FN episode (Fig. 1). Our primary outcome was any safety relevant event (SRE), defined as bacteremia and/or serious medical complications. Bacteremia was defined by the detection of a recognized pathogen from one or more blood cultures according to current definitions^19,20^. A serious medical complication was defined as death due to any cause during FN, admission to an intensive care unit (ICU), high dependency unit or other critical care unit for organ support, or severe sepsis (including septic shock) defined as sepsis plus a specified severe organ dysfunction, according to established definitions^14,21^. Length of hospital stay was measured in days from the date of hospitalization to discharge. Restarting antibiotics within 7 days and with persistent neutropenia was counted as the same FN episode, as long as intravenous chemotherapy had not been restarted^14^.

### Data quality

To enhance data accuracy, we manually checked the original paper CRFs for additional or wrongly transferred information if: (1) episodes had missing time points; (2) times exceeded 300 min; (3) episodes showed illogical sequence patterns during graphical assessment (e.g. contradictory negative time spans as start of antibiotics before arrival). We corrected 18 times that were wrongly transferred and added five missing times. To avoid a systematical bias due to an abnormal course of events, we excluded episodes with delays > 300 min because no neutropenia was suspected (n = 4) and episodes with delays of > 300 min for unknown reasons before calling or arrival at the hospital from all analyses (n = 5). Only for the analysis of two secondary time spans (38.5 °C to start and arrival to start of antibiotics), we also excluded episodes with early arrival and waiting to cross the current fever limit at the hospital (n = 67) or starting antibiotics with delay without reaching the current fever limit (n = 11).

### Statistical analyses

The statistical analysis plan was defined before the start of the analysis (Online Resource Text S1). We performed descriptive statistic using standard methods and missing data were not imputed. The association between TTA and outcomes was evaluated with linear and logistic three-level mixed regression with random intercepts per patient, nested within center. We categorized TTA in three different ways. First as a categorical variable with six predefined intervals: ≤ 30 min, 31–60 min, 61–120 min, 121–180 min, 181–240 min and > 240 min^11,22,23^. Secondly, we used the adjacent categories method^24,25^, applying three-level mixed logistic regression on SRE, to identify informative time intervals. We started with 17 categories (eight 15 min intervals from 0 to 120 min, and eight 30 min intervals from 120 to 360 min, the 17th category thus being > 360 min). We then combined the adjacent time categories with the least significant differences step by step, until differences between the combined categories were significantly different (*p*-value ≤ 0.2). The resulting categories were: ≤ 15 min, 16–150 min, 151–180 min, 181–240 min and > 240 min. At last, we analyzed TTA as binary variable ≤ 60 min versus > 60 min^8,11,13,26^.

For adjustment of triage bias, we developed three different propensity scores for TTA, balancing the probability of treatment assignment with observed baseline variables The first score included three variables that are clinically relevant to define a poor general condition at presentation (clinical reason to diagnose FN, systemic inflammatory response syndrome or severe sepsis at presentation) and three variables known to influence the risk of SRE (type of malignancy^27,28^, bone marrow involvement^27^ and leucocyte count^29^) (Online Resource Table S1). The second score included all 22 variables accessible at diagnosis of FN (Online Resource Table S1). The third score included those variables among the 22 that were significantly associated with TTA (*p*-value ≤ 0.1) in a three-level mixed linear regression model (Online Resource Table S1). Variables changing during treatment were re-assessed each month. To correct for the study design, we included the randomized fever limit and location at presentation in all three propensity scores, whether significant or not. We assigned the propensity scores as a continuous variable to each episode.

To analyze the association between TTA and SRE within different groups of patients, we performed three stratified analyses with potential effect modifiers (Fig. 1): (1) severe disease (defined as severe sepsis or reduced clinical condition (i.e., a general deterioration in physical health)) versus no severe disease at presentation with FN, assessed at first contact by the responsible physician; (2) location at FN diagnosis (patients present in the hospital versus outpatients); (3) first FN episode of a patient versus any later episode without prior SRE versus later episode with prior SRE.

We used two-sided tests and calculated 95% confidence intervals (CI). For all calculations we used R 4.0.5^30^. Specifically we used, the “glmer” function from the “lme4” library^31^ for mixed logistic regression and the “lme” function from the “nlme” library^32^ for mixed linear regression.

## Results

### Patients and episodes

**Figure 2:**
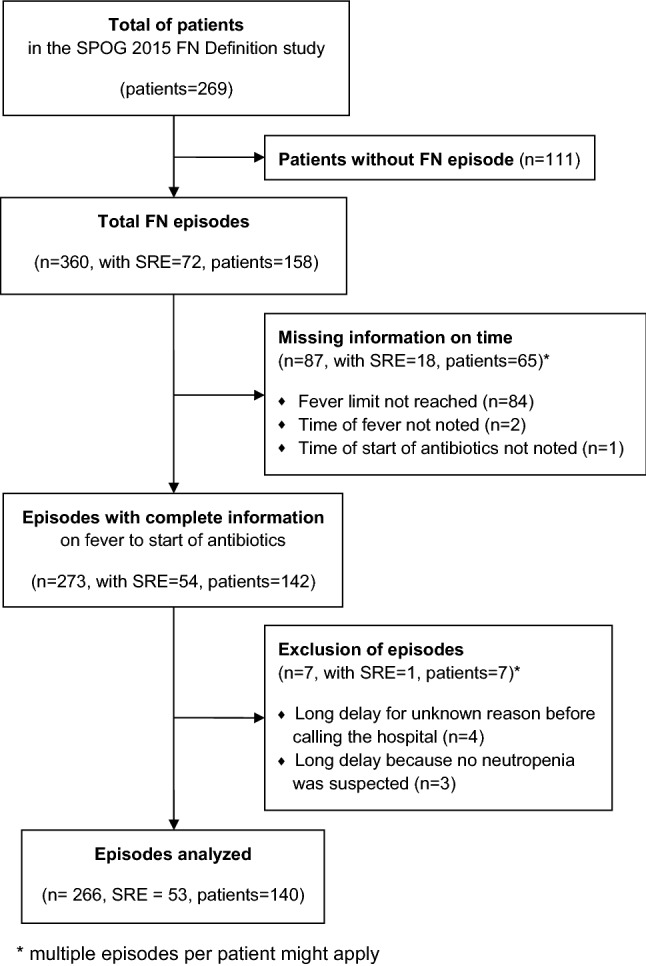
Flow chart of patients and episodes analyzed for fever (38.5 °C or 39.0 °C) to start of antibiotics (TTA).

**Figure 3:**
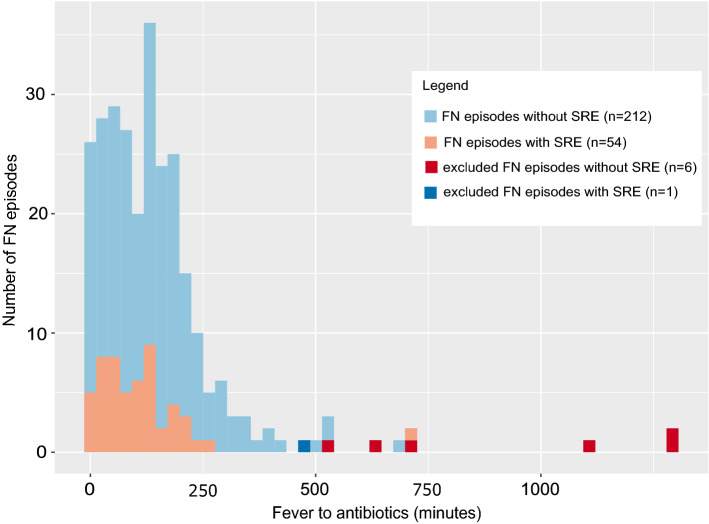
Distribution of FN episodes and safety relevant events according to fever (38.5 °C or 39.0 °C) to start of antibiotics (TTA).

The SPOG 2015 FN Definition study consecutively recruited 269 patients. Among them 158 (59%) developed a total of 360 FN episodes. For the main analysis of TTA, we included 266 (74%) FN episodes in 140 (89%) patients, including 53 (20%) with SRE (Figs. 2 and 3). Fifty-nine (42%) patients were female, median age was 6 (IQR 3–11) and acute lymphoblastic leukemia was the predominant diagnosis (Online Resource Table S2). Further details on the study population have been published^14^.

### Time from fever to start of antibiotics (TTA) and safety relevant events

The median TTA was 120 min (IQR 49–180, max 710), shorter in episodes with SRE (94 min) compared to episodes without (125 min). We did not find an association between TTA and the risk of SRE. If anything we found a trend for a lower risk of SRE with longer TTA (Table 1, Fig. 3).

**Table 1:** Association between time from fever to start of antibiotics (TTA) and the occurrence of safety relevant events (SRE).

TTA			Results of three-level mixed logistic regression without propensity score		With propensity score applied	
	FN episodes (% of 266)	SRE (% of 53)	Rate ratio (95% CI)	*p*-value	Rate ratio (95% CI)	*p*-value
**Predefined intervals**						
≤ 30 min	51 (19%)	13 (25%)	1 (Reference)	–	1 (Reference)	–
31–60 min	26 (10%)	6 (11%)	0.75 (0.22–2.62)	0.654	0.93 (0.25–3.38)	0.907
61–120 min	61 (23%)	15 (28%)	0.87 (0.34–2.25)	0.772	1.36 (0.48–3.84)	0.564
121–180 min	62 (23%)	10 (19%)	0.44 (0.15–1.25)	0.122	0.79 (0.24–2.53)	0.686
181–240 min	39 (15%)	7 (13%)	0.54 (0.18–1.68)	0.291	1.01 (0.28–3.58)	0.987
> 240 min	27 (10%)	2 (4%)	0.19 (0.04–1)	0.050	0.41 (0.07–2.51)	0.336
**Adjacent categories**						
≤ 15 min	34 (13%)	6 (11%)	1 (Reference)	–	1 (Reference)	–
16–150 min	145 (55%)	37 (70%)	1.60 (0.56–4.58)	0.378	2.49 (0.8–7.7)	0.114
151–180 min	21 (8%)	1 (2%)	0.19 (0.02–1.85)	0.153	0.32 (0.03–3.24)	0.332
181–240 min	39 (15%)	7 (13%)	0.96 (0.26–3.54)	0.954	1.92 (0.46–8.05)	0.373
> 240 min	27 (10%)	2 (4%)	0.33 (0.06–1.97)	0.226	0.80 (0.12–5.39)	0.817
**Binary variable**						
≤ 60 min	77 (29%)	19 (36%)	1 (Reference)	–	1 (Reference)	–
> 60 min	189 (71%)	34 (64%)	0.60 (0.29–1.22)	0.157	1.1 (0.46–2.45)	0.891

*CI* confidence interval, *FN* fever in neutropenia, *TTA* time from fever to start of antibiotics, *SRE* safety relevant event.

When we applied the first propensity score we saw a shift towards equal or non-significantly higher risk of SRE for longer TTA. (Table 1). The effect of the adjustment was smaller using the second or third propensity score. Therefore, we only kept the first propensity score for further analyses. We found comparable changes in effect size when we applied the propensity score in the second and third approach for categorizing time (adjacent categories method and ≤ 60 min versus > 60 min). Sensitivity analyses for TTA and SRE without exclusion of the 7 episodes with delays > 300 min revealed comparable results (Online Resource Table S3).

### Analysis by disease severity at presentation

**Figure 4:**
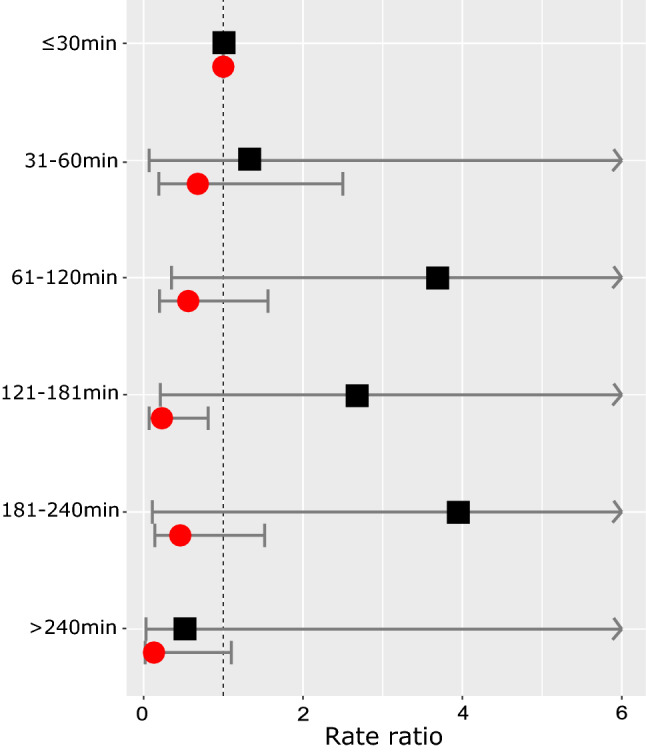
Results of three-level mixed logistic regression of fever (38.5 °C or 39.0 °C) to start of antibiotics (TTA) and safety relevant events, stratified by severity of the disease at presentation. Legend: Displayed are rate ratios and 95% confidence interval (x-axis) for the predefined time intervals (y-axis). Black squares indicate rate ratios of patients with severe disease at presentation (defined as severe sepsis or reduced clinical condition); red circles indicate rate ratios from patients without severe disease at presentation.

Severe disease at presentation was recorded in 36 (14%) of the 266 episodes, with a median TTA of 120 min (IQR 59–162). In the remaining 230 (86%) episodes without severe disease at presentation, median TTA was also 120 min (IQR 49–185). In patients presenting with severe disease, we found a trend for an association of longer TTA with higher risk of SRE (Table 2, Fig. 4). The propensity score was not applied here due to overlapping definitions of severe disease and variables included in the score.

**Table 2:** Stratified analysis according to severity of disease at presentation for the association of time from fever to start of antibiotics (TTA) and the occurrence of safety relevant events (SRE).

			Results of three-level mixed logistic regression without propensity score	
	FN episodes	SRE	Rate ratio (95% CI)	*p*-value
**Predefined intervals**				
Severe disease at presentation	36 (100%)	20 (100%)		
≤ 30 min	5 (14%)	2 (10%)	1 (Reference)	–
31–60 min	4 (11%)	2 (10%)	1.33 (0.07–24.48)	0.849
61–120 min	10 (28%)	7 (35%)	3.69 (0.35–38.68)	0.276
121–180 min	10 (28%)	6 (30%)	2.68 (0.21–33.86)	0.446
181–240 min	3 (8%)	2 (10%)	3.95 (0.11–137.6)	0.448
> 240 min	4 (11%)	1 (5%)	0.52 (0.03–9.9)	0.661
No severe disease at presentation	230 (100%)	33 (100%)		
≤ 30 min	46 (20%)	11 (33%)	1 (Reference)	–
31–60 min	22 (10%)	4 (12%)	0.68 (0.19–2.5)	0.563
61–120 min	51 (22%)	8 (24%)	0.56 (0.2–1.56)	0.263
121–180 min	52 (23%)	4 (12%)	0.23 (0.07–0.81)	0.022
181–240 min	36 (16%)	5 (15%)	0.46 (0.14–1.52)	0.204
> 240 min	23 (10%)	1 (3%)	0.13 (0.02–1.1)	0.061
**Binary variable**				
Severe disease at presentation	36 (100%)	20 (100%)		
≤ 60 min	9 (25%)	4 (20%)	1 (Reference)	–
> 60 min	27 (75%)	16 (80%)	2.02 (0.34–12.06)	0.440
No severe disease at presentation	230 (100%)	33 (100%)		
≤ 60 min	68 (30%)	15 (45%)	1 (Reference)	–
> 60 min	162 (70%)	18 (55%)	0.41 (0.19–0.88)	0.023

*CI* confidence interval, *FN* fever in neutropenia, *TTA* time from fever to start of antibiotics, *SRE* safety relevant event.

### Sensitivity analyses

We repeated the analysis for secondary outcomes (serious medical complication, bacteremia and length of hospital stay) and secondary time spans. Both approaches had similar results as the main analysis.

Regarding secondary outcomes, in 19 (7%) of 266 episodes, at least one serious medical complication was reported (zero death, 9 ICU admissions and 15 severe sepsis), median TTA was 96 min (IQR 43–138 min, max 225 min). Bacteremia occurred in 42 (16%) episodes, median TTA was 86 min (IQR 30–135 min, max 710 min). Median length of hospital stay was 5 days (IQR 4–10 days, max 78 days), it was longer in patients with bacteremia, serious medical complications and SRE. Results for secondary outcomes were comparable to results for the primary outcome (Online Resource Table S4).

Regarding secondary time spans, time from first temperature measurement of 38.5 °C to start of antibiotics was analyzed in 227 (63%) of 360 episodes, median time was 145 min (IQR 87–210 min, max 1405 min). Time before arrival at the hospital was analyzed in 237 (66%) episodes, median time to arrival was 60 min (IQR 0–110 min, max 1396 min). Time after arrival to start of antibiotics was analyzed in 263 (73%) episodes, median time was 70 min (IQR 45–120 min, max 633 min). Results for secondary time spans were comparable to those of the primary TTA (Online Resource Table S5).

Stratification for location at FN diagnosis showed a clearer trend for a lower risk of SRE with longer TTA in patients not at study site versus patients at study site, with little change when applying the propensity score (Online Resource Table S6). We found no differences between first versus later FN episodes with or without prior SRE.

## Discussion

Overall, this analysis did not provide evidence that a longer time span between recognition of fever and start of antibiotics leads to a higher risk for a poor clinical outcome. On the contrary, patients who developed an SRE arrived earlier at the hospital and received antibiotics faster than those who had no SRE. This counterintuitive result of poorer outcomes for shorter TTA is most likely due to confounding by the clinical condition of the child, the so called triage bias^7^. Triage reflects prioritization of presentation, assessment and treatment, and is influenced by both parents and hospital staff. Applying our propensity score reduced the size of this bias. Only among patients with severe disease at presentation there was a trend for higher risk of SRE with longer TTA.

Strengths of this analysis are its prospective, multicenter design, the representative study population including a broad spectrum of malignancies and the standardized temperature measurement. We had few missing data and were also aware of the lag time before the patients’ arrival at the hospital. Methodological strengths were the attempt to adjust for triage bias and the use of several pre-defined intervals instead of just splitting TTA at 1 h.

Results are limited by the low number of FN episodes with SRE, especially in stratified analyses and, when applied to patients with a specific malignancy, resulting in limited precision and statistical power. Although we corrected for multiple episodes per patients, different centers and the two randomized fever limits used, we cannot exclude a potential interference of the randomized controlled study design on our results. The relatively short travel times to the hospital of a maximum around 3 h, may affect generalizability of these results to bigger countries. The predefined primary outcome SRE includes episodes with bacteremia. Delay in antibiotic treatment may allow dissemination of an infection, leading ultimately to bacteremia, but bacteremia can, of course, also occur independently of TTA. However, comparable results in secondary analysis of serious medical complications and of bacteremia increase the credibility of our findings.

Most previous studies that investigated TTA in pediatric patients under chemotherapy, found no association between longer TTA and death^12,13,26^ admission to the ICU^11,13^ or bacteremia^11,26^. This is comparable to our results. Only one retrospective study reported an association with more adverse events (in-hospital mortality, admission to ICU, receipt of ≥ 40 ml/kg fluid resuscitation) for TTA of 61–120 min versus ≤ 60 min^11^. A recent prospective Australian study found a significantly shorter length of stay in the hospital in patients with TTA > 60 min (*p* = 0.016)^33^, a finding we did not reproduce.

Assessment of a patient’s clinical condition is often subjective, a reality we tried to capture by including the variable “reduced clinical condition” into the definition for severe disease. Our results suggest that patients presenting with severe disease may be the ones who profit from short TTA. This is in contrast to the report of Fletcher et al.^11^, who suggested that TTA may not have an impact in patients presenting with severe sepsis, as they are likely to have a poor outcome irrespective of TTA. We hypothesize that patients presenting without severe disease may include two groups of patients: those in whom antibiotics are needed and a short TTA is beneficial; and those who do not need antibiotics because of fever due to viral infections, hence a short TTA is not influencing outcome.

Triage bias was present in our analysis and adjustment for this bias with a propensity score changed the association in the direction expected. For patients not at study site triage bias seems to be even more important. Two prospective observational studies in pediatric patients reported that patients with sepsis received antibiotics sooner than those without sepsis, indicating the relevance of triage bias^12,13^. In both these studies the time of sepsis assessment is not reported.

The ideal design for the direct investigation of TTA on FN outcome would be a randomized controlled trial. From an ethical point of view, such a study does not seem to be possible. But future observational clinical studies might aim to still better assess variables reflecting triage bias and may improve evidence when combined with meta-analysis, be it conventional or individual patient data based meta-analysis. This may include additional laboratory parameters, results of rapid microbiological tests, clinical parameters related to sepsis or severe infection, information on outcomes of past FN episodes, and information available before arrival at the hospital. Future studies might also focus on distinction of patients at different risks for poor outcome, and may even consider to only include patients presenting with severe disease.

Multiple studies have shown that time from arrival at the hospital to start of empirical antibiotics can effectively be shortened with different interventions^9^. After our analysis it is still unclear whether shorter TTA actually improves outcome in pediatric FN and no clear cut-off can be recommended. Though, timely administration of antibiotics may be desirable as element of care, but it may not be a suitable metric for quality assessments. Resources used to reduce TTA may be better spent on exact clinical evaluation and regulations on patient’s location during chemotherapy have to be reflected carefully. In patients presenting without severe disease, reproduction of our findings would support the consideration to relax metrics for rapid administration of antibiotics as they may not be beneficial and could even result in unnecessary harm like side effects and development of resistances.

## Conclusion

Overall, in this analysis of prospectively collected data in pediatric patients with FN undergoing chemotherapy for cancer, TTA was unrelated to poor clinical outcome in pediatric patients with FN presenting without severe disease. Only in patients with severe disease at presentation we found a trend for the expected improvement in outcomes with shorter TTA. We found strong evidence for a triage bias, influencing both time to arrival and time to antibiotics. Adjustment for this triage bias by applying a propensity score was possible to a certain extent, but needs further improvement.

## Supplementary Information

Supplementary Information.

## Data Availability

The trial protocol is available on https://www.spog.ch/wp-content/uploads/2020/03/SPOG_FN_Protocol1.1_20161123_PDF.pdf. The statistical analysis plan for the analysis presented in this manuscript is available in the supplementary information (Online Resource Text S1). Participant data are available for qualified researchers who wish to access the data following article publication; no end data. Proposals should be directed to roland.ammann@insel.ch; to gain access, data requestors will need to sign a data access agreement.
