# Supplementary material for: Time to antibiotics is unrelated to outcome in pediatric patients with fever in neutropenia presenting without severe disease during chemotherapy for cancer
Source: Sci Rep. 2022 Aug 18;12:14028. doi: 10.1038/s41598-022-18168-x (PMC9388602; doi:10.1038/s41598-022-18168-x)
Supplement: Supplementary file 1 — Supplementary Information. [file 41598_2022_18168_MOESM1_ESM.pdf]

Supplementary material

Time to antibiotics is unrelated to outcome in pediatric patients with fever in neutropenia during chemotherapy for cancer presenting without severe disease

Christa Koenig, Claudia E Kuehni, Nicole Bodmer, Philipp KA Agyeman, Marc Ansari M, Jochen Roessler, Nicolas X von der Weid, Roland A Ammann

Contents

Online Resource Table S1 Propensity scores. 2

Online Resource Table S2 Patient characteristics. 3

Online Resource Table S3 Analysis without exclusion of episodes with delays >300min, for the association between time from fever to start of antibiotics (TTA) and the occurrence of safety relevant events (SRE). 4

Online Resource Table S4 Association between time from fever to start of antibiotics (TTA) and the occurrence of secondary outcomes in predefined intervals. 5

Online Resource Table S5 Association between secondary time spans and the occurrence of safety relevant events (SRE). 6

Online Resource Table S6 Stratified analysis according to location at FN diagnosis for the association of time from fever to start of antibiotics (TTA) and the occurrence of safety relevant events (SRE). ... 7

Online Resource Text S1 - Plan of Analysis 8

Online Resource Table S1 Propensity scores.

	First propensity score		Third propensity Score	
	Coefficient*	p-value	Coefficient[a]	p-value
**Fever limit**				
38.5°C	Reference	-	Reference	-
39.0°C	-2	0.104	-6	0.006
**Location**				
At study site	Reference	-	Reference	-
Not at study site	8	<0.001	5	0.020
**Time since diagnosis**				
<1 months	-	-	-9	0.004
1-8 months	-	-	-8	0.001
>8 months	-	-	-	-
**Max fever at diagnosis**				
<39.0°C	-	-	Reference	-
≥39.0°C	-	-	5	0.017
**Type of malignancy**				
Acute lymphoblastic leukemia	Reference	-	Reference	-
Acute myeloid leukemia	-5	0.221	-2	0.773
Hodgkin lymphoma	0	0.931	2	0.658
Non-Hodgkin lymphoma	-5	0.040	-4	0.162
Central nervous system tumour	3	0.238	7	0.086
Other solid tumour	-2	0.384	1	0.593
**Neutrophils**				
≥0.1G/l	-	-	Reference	-
<0.1G/l	-	-	-3	0.052
**Clinical reason for diagnosis**				
No	Reference	-	-	-
Yes	3	0.110	-	-
**SIRS at presentation**				
No	Reference	-	-	-
Yes	-2	0.357	-	-
**Sepsis at presentation**				
No	Reference	-	-	-
Yes	-3	0.293	-	-
**Bone marrow involvement**				
No	Reference	-	-	-
Yes	-3	0.237	-	-
**Leucocytes**				
≥0.3G/l	Reference	-	-	-
<0.3G/l	-2	0.075	-	-
	Score with 22 levels, -11 to 10		Score with 32 levels, 21 to 11	

The **second propensity score** had 41 levels, ranging from -32 to 17.
Variables used were: clinical reason to diagnose FN, systemic inflammatory response syndrome (SIRS) at presentation, severe sepsis at presentation, type of malignancy, bone marrow involvement, leucocyte count<0.3G/l, absolute neutrophil count <0.1G/l, haemoglobin ≥90g/l, platelet count <50G/l, sex, age at screening, chemotherapy intensity, time since diagnosis, presence of any central venous access device, relapse status, prior episodes of FN, prior episodes of FN with bacteraemia, prior episodes of FN with SRE, time of presentation (weekend vs. weekday and office time vs. out-of-office time), season (spring/summer vs. autumn/winter), fever ≥39.0°C at FN diagnosis, current fever limit and location at presentation.

[a] The coefficient used equals the respective coefficient from the three-level mixed linear regression model divided by 10 and rounded to the next integer.

Online Resource Table S2 Patient characteristics.

	Total patients in the SPOG 2015 FN Definition study	Patients with FN episodes	Patients studied for analysis of TTA
Number of patients	269 (100%)	158 (100%)	140 (100%)
Age at screening, years	8 (4 to 13)	6 (3 to 11)	6 (3 to 11)
Sex			
Female	105 (39%)	69 (44%)	59 (42%)
Male	164 (61%)	89 (56%)	81 (58%)
Type of malignancy			
Acute lymphoblastic leukemia	115 (43%)	70 (44%)	60 (43%)
Acute myeloid leukemia	7 (3%)	6 (4%)	6 (4%)
Hodgkin lymphoma	20 (7%)	6 (4%)	5 (4%)
Non-Hodgkin lymphoma	31 (12%)	18 (11%)	18 (13%)
Central nervous system tumor	76 (28%)	16 (10%)	15 (11%)
Other solid tumors		42 (27%)	36 (26%)
Center			
1	20 (7%)	13 (8%)	10 (7%)
2	78 (29%)	54 (34%)	48 (34%)
3	31 (12%)	19 (12%)	17 (12%)
4	35 (13%)	19 (12%)	16 (11%)
5	15 (6%)	9 (6%)	6 (4%)
6	90 (33%)	44 (28%)	43 (31%)

Data are median (IQR) or number (%). Percentages may not total 100 because of rounding.
Abbreviations: FN, fever in neutropenia; TTA, time from fever to start of antibiotics; IQR, interquartile range.

Online Resource Table S3 Analysis without exclusion of episodes with delays >300min, for the association between time from fever to start of antibiotics (TTA) and the occurrence of safety relevant events (SRE).

			**Results of three-level mixed logistic regression without propensity score**		**With propensity score applied**	
**TTA**	**FN episodes** (% of 273)	**SRE** (% of 54)	**Rate ratio (95% CI)**	**P-value**	**Rate ratio (95% CI)**	**P-value**
**Predefined intervals**						
≤30min	51 (19%)	13 (24%)	1 (Reference)		1 (Reference)	-
31-60min	26 (10%)	6 (11%)	0.78 (0.22 to 2.75)	0.694	0.95 (0.26 to 3.52)	0.942
61-120min	61 (22%)	15 (28%)	0.88 (0.33 to 2.30)	0.790	1.31 (0.46 to 3.74)	0.614
121 - 180min	62 (23%)	10 (19%)	0.44 (0.15 to 1.29)	0.135	0.77 (0.24 to 2.49)	0.657
181 - 240min	39 (14%)	7 (13%)	0.55 (0.18 to 1.74)	0.312	0.98 (0.27 to 3.48)	0.969
>240min	34 (12%)	3 (6%)	0.24 (0.06 to 1.02)	0.053	0.48 (0.10 to 2.32)	0.358
**Adjacent categories**						
≤15min	34 (12%)	6 (11%)	1 (Reference)	-	1 (Reference)	-
16-150min	145 (53%)	37 (69%)	1.64 (0.56 to 4.76)	0.366	2.51 (0.80 to 7.91)	0.117
151-180min	21 (8%)	1 (2%)	0.20 (0.02 to 1.95)	0.165	0.31 (0.03 to 3.26)	0.331
>180min	73 (27%)	10 (19%)	0.71 (0.21 to 2.38)	0.579	1.47 (0.38 to 5.72)	0.578
**Binary variable**						
≤60min	77 (28%)	19 (35%)	1 (Reference)	-	1 (Reference)	-
>60min	196 (72%)	35 (65%)	0.60 (0.29 to 1.23)	0.160	1.12 (0.48 to 2.63)	0.791

Abbreviations: CI, confidence interval; FN, fever in neutropenia; TTA, time from fever to start of antibiotics; IQR, interquartile range; SRE, safety relevant event.

Online Resource Table S4 Association between time from fever to start of antibiotics (TTA) and the occurrence of secondary outcomes in predefined intervals.

			**Results of three-level mixed logistic regression without propensity score**		**With propensity score applied**	
	**FN episodes** (% of 266)	**Serious medical complication** (% of 19)	Rate ratio (95% CI)	P-value	Rate ratio (95% CI)	P-value
≤30min	51 (19%)	4 (21%)	1 (Reference)	-	1 (Reference)	-
31-60min	26 (10%)	2 (11%)	1.08 (0.03 to 36.9)	0.966	1.65 (0.05 to 53.8)	0.779
61-120min	61 (23%)	7 (37%)	3.13 (0.26 to 38.0)	0.370	5.64 (0.34 to 93.4)	0.227
121 - 180min	62 (23%)	4 (21%)	1.88 (0.1 to 35.3)	0.674	3.58 (0.14 to 92.5)	0.442
181 - 240min	39 (15%)	2 (11%)	0.13 (0 to 8.93)	0.340	0.42 (0.01 to 30.1)	0.691
>240min	27 (10%)	0 (0%)	-	-	-	-
			**Results of three-level mixed logistic regression without propensity score**		**With propensity score applied**	
	**FN episodes** (% of 266)	**Bacteraemia** (% of 42)	Rate ratio (95% CI)	P-value	Rate ratio (95% CI)	P-value
≤30min	51 (19%)	12 (29%)	1 (Reference)	-	1 (Reference)	-
31-60min	26 (10%)	5 (12%)	0.64 (0.18 to 2.34)	0.505	0.86 (0.22 to 3.32)	0.821
61-120min	61 (23%)	10 (24%)	0.58 (0.21 to 1.57)	0.280	0.97 (0.33 to 2.86)	0.952
121 - 180min	62 (23%)	7 (17%)	0.34 (0.11 to 1.06)	0.063	0.68 (0.19 to 2.38)	0.544
181 - 240min	39 (15%)	6 (14%)	0.54 (0.17 to 1.67)	0.284	1.09 (0.3 to 3.96)	0.893
>240min	27 (10%)	2 (5%)	0.23 (0.04 to 1.18)	0.078	0.56 (0.09 to 3.4)	0.533
	**Length of hospital stay**		**Results of three-level mixed linear regression without propensity score**		**With propensity score applied**	
	Median [days]	IQR (max) [days]	Coefficient (95% CI)	P-value	Coefficient (95% CI)	P-value
≤30min	6	4.5 to 11.0 (78)	1 (Reference)	-	1 (Reference)	-
31-60min	7.5	4.3 to 13.8 (27)	-0.3 (-4.5 to 3.8)	0.869	0.6 (-3.5 to 4.7)	0.777
61-120min	6	4.0 to 10.0 (77)	-0.9 (-4.1 to 2.3)	0.587	0.6 (-2.8 to 4.0)	0.718
121 - 180min	4	3.0 to 6.0 (32)	-3.8 (-7.0 to -0.5)	0.027	-1.7 (-5.3 to 1.8)	0.343
181 - 240min	5	4.0 to 11.0 (35)	-1.8 (-5.4 to 1.8)	0.327	0.3 (-3.5 to 4.1)	0.875
>240min	5	3.5 to 10.0 (17)	-3.5 (-7.6 to 0.6)	0.099	-0.8 (-5.4 to 3.7)	0.718

Abbreviations: CI, confidence interval; FN, fever in neutropenia; TTA, time from fever to start of antibiotics; IQR, interquartile range.

Online Resource Table S5 Association between secondary time spans and the occurrence of safety relevant events (SRE).

**Time span**			**Results of three-level mixed logistic regression without propensity score**		**With propensity score applied**	
**38.5°C to start of antibiotics**	**FN episodes** (% of 227)	**SRE** (% of 49)	Rate ratio (95% CI)	P-value	Rate ratio (95% CI)	P-value
≤30min	27 (12%)	10 (20%)	1 (Reference)	-	1 (Reference)	-
31-60min	13 (6%)	6 (12%)	1.42 (0.28 to 7.09)	0.672	1.51 (0.31 to 7.33)	0.611
61-120min	47 (21%)	11 (22%)	0.47 (0.14 to 1.52)	0.205	0.59 (0.18 to 1.94)	0.382
121 - 180min	63 (28%)	10 (20%)	0.26 (0.08 to 0.88)	0.030	0.42 (0.11 to 1.59)	0.202
181 - 240min	41 (18%)	8 (16%)	0.36 (0.10 to 1.28)	0.116	0.58 (0.15 to 2.27)	0.436
>240min	36 (16%)	4 (8%)	0.17 (0.04 to 0.75)	0.019	0.29 (0.06 to 1.39)	0.122

			**Results of three-level mixed logistic regression without propensity score**		**With propensity score applied**	
**38.5°C to arrival at the hospital**	**FN episodes** (% of 237)	**SRE** (% of 50)	Rate ratio (95% CI)	P-value	Rate ratio (95% CI)	P-value
≤30min						
31-60min	80 (34%)	20 (40%)	1 (Reference)	-	1 (Reference)	-
61-120min	39 (16%)	11 (22%)	1.21 (0.48 to 3.04)	0.680	1.98 (0.73 to 5.40)	0.182
121 - 180min	71 (30%)	15 (30%)	0.81 (0.36 to 1.83)	0.620	1.45 (0.58 to 3.67)	0.427
181 - 240min	26 (11%)	2 (4%)	0.23 (0.05 to 1.08)	0.063	0.47 (0.09 to 2.49)	0.376
>240min	7 (3%)	1 (2%)	0.51 (0.05 to 4.81)	0.557	0.90 (0.09 to 9.14)	0.929
	14 (6%)	1 (2%)	0.20 (0.02 to 1.69)	0.140	0.31 (0.03 to 2.66)	0.283

			**Results of three-level mixed logistic regression without propensity score**		**With propensity score applied**	
**Arrival at the hospital to start of antibiotics**	**FN episodes** (% of 263)	**SRE** (% of 58)	Rate ratio (95% CI)	P-value	Rate ratio (95% CI)	P-value
≤30min	51 (19%)	16 (28%)	1 (Reference)	-	1 (Reference)	-
31-60min	54 (21%)	15 (26%)	0.72 (0.26 to 2.01)	0.536	0.91 (0.32 to 2.60)	0.855
61-120min	93 (35%)	18 (30%)	0.49 (0.19 to 1.25)	0.135	0.67 (0.25 to 1.80)	0.424
121 - 180min	37 (14%)	5 (9%)	0.32 (0.09 to 1.13)	0.076	0.44 (0.12 to 1.62)	0.215
181 - 240min	17 (6%)	2 (3%)	0.19 (0.03 to 1.18)	0.075	0.27 (0.04 to 1.68)	0.160
>240min	11 (4%)	2 (3%)	0.39 (0.05 to 2.60)	0.333	0.54 (0.08 to 3.68)	0.531

Abbreviations: CI, confidence interval; FN, fever in neutropenia; IQR, interquartile range; SRE, safety relevant event.

Online Resource Table S6 Stratified analysis according to location at FN diagnosis for the association of time from fever to start of antibiotics (TTA) and the occurrence of safety relevant events (SRE).

**Location at FN diagnosis**			**Results of three-level mixed logistic regression without propensity score**		**With propensity score applied**	
**Not at study site**	**FN episodes** (% of 178)	**SRE** (% of 31)	Rate ratio (95% CI)	p-value	Rate ratio (95% CI)	p-value
<=30min	15 (%)	4 (%)	1 (Reference)	-	1 (Reference)0.22 (0.01 to 3.28)	-
31-60min	10 (%)	1 (%)	0.25 (0.02 to 2.88)	0.264	0.37 (0.07 to 2.15)	0.271
61-120min	45 (%)	10 (%)	0.49 (0.1 to 2.31)	0.369	0.39 (0.07 to 2.36)	0.271
121 - 180min	52 (%)	9 (%)	0.35 (0.07 to 1.73)	0.198	0.45 (0.08 to 2.65)	0.308
181 - 240min	33 (%)	6 (%)	0.39 (0.08 to 1.96)	0.252	0.15 (0.01 to 2)	0.377
>=240min	23 (%)	1 (%)	0.08 (0.01 to 0.9)	0.041		0.151
**At study site**	**FN episodes** (% of 88)	**SRE** (% of 22)	Rate ratio (95% CI)	p-value	Rate ratio (95% CI)	p-value
<=30min	36 (%)	9 (%)	1 (Reference)	-	1 (Reference)	-
31-60min	16 (%)	5 (%)	1.54 (0.3 to 8.03)	0.608	2.1 (0.28 to 15.49)	0.469
61-120min	16 (%)	5 (%)	1.42 (0.3 to 6.8)	0.663	2.06 (0.32 to 13.68)	0.456
121 - 180min	10 (%)	1 (%)	0.29 (0.03 to 3.41)	0.327	0.35 (0.03 to 4.62)	0.423
181 - 240min	6 (%)	1 (%)	0.56 (0.04 to 7.78)	0.667	0.71 (0.04 to 12.82)	0.819
>=240min	4 (%)	1 (%)	1 (0.06 to 17.34)	0.999	1.63 (0.07 to 41.03)	0.765

Abbreviations: CI, confidence interval; FN, fever in neutropenia; IQR, interquartile range; SRE, safety relevant event.

Online Resource Text S1 - Plan of Analysis

Version 1.1. of June 6, 2021. See Appendix for changes from the plan of analysis version 1.0 of February 11, 2021.

Association of time to antibiotics (TTA) with safety relevant events (SRE) in children undergoing chemotherapy for cancer with fever in neutropenia (FN) – SPOG 2015 FN Definition study (NCT02324231)

Data used was collected during the SPOG 2015 FN Definition study, a randomized controlled non-blinded multicenter trial (including 360 fever in neutropenia (FN) episodes). It primarily aimed to determine if a fever limit of 39.0°C is non-inferior to 38.5°C regarding safety in children and adolescents with cancer treated with chemotherapy. FN was diagnosed at temperatures reaching the current fever limit (38.5°C or 39.0°C), but diagnosis below this limit was allowed if clinically indicated.[1,2]

1) Aims and Questions

1.1) Primary aim

The main question we want to address with this analysis is: Is there an association between time to antibiotics (TTA) and safety relevant events (SRE) in children undergoing chemotherapy for cancer with FN? We want to know if TTA is influencing the amount of SREs, and if there is a specific threshold for TTA.

1.2) Correction for triage and other biases

We aim to correct for these biases by creating a propensity score (2.2).

1.3) Secondary aims

Secondary questions are:

Is there an association between TTA and other clinical outcomes?
Is there a difference in the association with SRE for the time before arrival at the hospital (reaching 38.5°C to arrival) and after (arrival to start of antibiotics?)
Is there only an association of TTA and SRE when the fever limit used for diagnosis is reached?
Is our data supporting the "golden hour" rule, i.e. to administer antibiotics within one hour after arrival?
What is the influence of location at diagnosis, condition at presentation, outcome of previous FN episodes and the fever limit used for FN diagnosis on the association of TTA and SRE?

2) Description of analyses

Descriptive statistics using standard methods will be performed.

2.1) Primary analysis

The primary analysis will be on the association between TTA (reaching the current fever limit (38.5°C or 39.0°C) to start of antibiotics) and episodes with a safety relevant event (SRE), corrected for variables influencing TTA with a propensity score.

Specific Aims/Questions:

- Is there an association between time to antibiotics (TTA) and safety relevant events (SRE)?
- Is there a specific threshold for TTA?
- What is the impact of biases on the association between TTA and SRE?

Analysis:

Three-level mixed logistic regression will be done with random intercepts per patient, nested within center to account for multiple episodes within patients.

First TTA will be analyzed as categorical variable with six categories that have been shown to be important in previous studies[3-5]: (≤30min / 31-60min / 61 – 120min / 121 – 180 / 181- 240 / ≥240)   240)

Second, if, by eyeballing, this first analysis is compatible with a linear or cubic association, the corresponding analysis of TTA as continuous variable will be tested against the first analysis using a likelihood ratio test.

Third, to answer the threshold question, the adjacent categories method will be used to identify meaningful time intervals, as the cut-off for more or less SRE, or for an association of TTA with SRE, may not be within the predefined time intervals. At start of this analysis intervals of 15min will be used up to 120min and 30min intervals up to 360min.

Correction for biases

These three analyses will be performed without and with adjustment for the propensity scores constructed under 2.2.

2.2) Creation of propensity scores

Patients presenting in reduced general condition or suspected to have a high risk for complications may receive treatment faster, but still have a potentially worse outcome. On the other hand, in patients at low risk for complications and in good general condition, treatment may be delayed, but the outcome is still good. This is creating a triage bias. Surely triage bias can influence time from arrival to start of antibiotics. We hypothesize that it may also already be important before arrival, because parents present faster when their child looks severely ill. In addition, other variables known at presentation may effect TTA and SRE.

Analysis:

Three different propensity scores will be developed using three level mixed linear regression models.

A) Propensity score for triage bias (restricted)

Using only variables that are clinically evident or known to influence the risk for SRE.

Clinically evident variables of poor condition:

- Clinical reason to diagnose FN (other reason / severely reduced condition / no)
- Systemic inflammatory response syndrome (SIRS) at presentation (yes/no)
- Severe sepsis at presentation (yes/no)

Variables known to influence the risk for SRE in our data and other studies:

- Type of malignancy (ALL, AML, HL, NHL, CNS tumor, other solid tumors)[6,7]
- Bone marrow involvement (yes/no)[6]
- Leukocyte count (<0.3G/l / ≥0.3G/l)[8]
- *Fever limit (38.5°C / 39.0°C)
- * Location at FN diagnosis (at study site / not at study site)

B) Propensity score for any variables known at presentation (inclusive)

All variables available and known at presentation, for which an influence on TTA is not excluded.

Variables assessed:

- Sex
- Age at screening (1 to 4 years / 5 to 8 years / 9 to 12 years / ≥ 13 years)[9]
- Type of malignancy (ALL, AML, HL, NHL, CNS tumor, other solid tumors)[6,7]
- Chemotherapy intensity (1 / 2-4)[8]
- Time since diagnosis (<1month / 1-7months / >8months)
- Bone marrow involvement (yes/no)[6]
- Presence of any central venous access device (yes/no)[6,9]
- Relapse status (yes/no)[10]
- Prior episodes of FN (yes/no)[5]
- Prior episodes of FN with bacteremia (yes/no)
- Prior episodes of FN with SRE (yes/no)
- Time of presentation (weekend / weekday and office time / out-of-office time)
- Season (Spring/summer (March to August) vs. autumn/winter (Sept. to February)
- Clinical reason to diagnose FN (other reason / severely reduced condition / no)
- SIRS at presentation (yes/no)
- Severe sepsis at presentation (yes/no)
- Absolute neutrophil count (<0.1G/l / ≥0.1G/l)[5,11,12]
- Hemoglobin (<90g/l / ≥90g/l)[8,13]
- Platelet count (<50G/l / ≥50G/l)[8,11,13,14]
- Leukocyte count (<0.3G/l / ≥0.3G/l)[8]
- Fever >= 39.0° at FN diagnosis (irrespective of current fever limit)[11,12,15]
-* Fever limit (38.5°C / 39.0°C)
- * Location at FN diagnosis (at study site / not at study site)

C) *Propensity score with significantly associated variables*

Univariable three-level mixed linear regression analysis will be performed for continuous TTA (fever to start of antibiotics). Variables significantly associated with TTA will be used for multivariable analysis (stepwise procedure, p≤0.10 for entry and for removal). *Fever limit (38.5°C vs. 39.0°C) and location at FN diagnosis will be kept in the model, whether significant or not, to adjust for the study design.

The coefficient of variables in the multivariable models will be used to create the propensity score. The score will be assigned to each episode.

2.3) Secondary analyses

a) Secondary analysis comparable to the primary analysis will additionally be done for three additional timespans and outcomes:

Timespans:
1) Reaching 38.5°C to arrival at the hospital
2) Arrival at the hospital to start of antibiotics
3) Reaching 38.5°C to start of antibiotics

Outcomes:
1) Serious medical complications (binary outcome)
2) Bacteremia (binary outcome)
3) Length of stay in the hospital (continuous outcome).

b) Three level mixed logistic regression will be performed for TTA (arrival to start of antibiotics) as binary variable (≤60min versus >60min (binary).

c) Stratified analysis will be done comparable to the primary analysis with the following variables:

1) Location at FN diagnosis: Inpatients or outpatient department versus not in study site (e.g. at home, in school)
2) With severe sepsis or reduced condition at presentation vs. without
3) First episode per patient versus any later episode with previous SRE versus any later episode without previous SRE

2.4) Exclusion of episodes

Descriptive analyses of the primary analysis will first done with all available episodes.

Then and for all the following analyses, episodes with early arrival and waiting to cross the current fever limit at the hospital (n=67) or starting antibiotics with delay without reaching the current fever limit (n=11) will be excluded from all analyses with the timespans:

1) Reaching 38.5°C to start of antibiotics

2) Arrival in the hospital to start of antibiotics

In addition, episodes with long delays because the patient were thought to be non-neutropenic (n=4) and episodes with long waiting for unknown reasons before calling the hospital (n=5) will be excluded from analysis of all TTAs.

References

1. Koenig C, Bodmer N, Agyeman PKA, et al. 39.0 degrees C versus 38.5 degrees C ear temperature as fever limit in children with neutropenia undergoing chemotherapy for cancer: a multicentre, cluster-randomised, multiple-crossover, non-inferiority trial. Lancet Child Adolesc Health 2020;4:495-502.

2. SPOG 2015 FN Definition Protocol 2016. at https://www.spog.ch/wp-content/uploads/2020/03/SPOG_FN_Protocol1.1_20161123_PDF.pdf.

3. Rosa RG, Goldani LZ. Cohort study of the impact of time to antibiotic administration on mortality in patients with febrile neutropenia. Antimicrob Agents Chemother 2014;58:3799-803.

4. Fletcher M, Hodgkiss H, Zhang S, et al. Prompt administration of antibiotics is associated with improved outcomes in febrile neutropenia in children with cancer. Pediatr Blood Cancer 2013;60:1299-306.

5. Daniels LM, Durani U, Barreto JN, et al. Impact of time to antibiotic on hospital stay, intensive care unit admission, and mortality in febrile neutropenia. Support Care Cancer 2019;27:4171-4177.

6. Ammann RA, Hirt A, Luthy AR, Aebi C. Identification of children presenting with fever in chemotherapy-induced neutropenia at low risk for severe bacterial infection. Med Pediatr Oncol 2003;41:436-43.

7. Phillips RS, Sung L, Ammann RA, et al. Predicting microbiologically defined infection in febrile neutropenic episodes in children: global individual participant data multivariable meta-analysis. Br J Cancer 2016;114:e17.

8. Ammann RA, Bodmer N, Hirt A, et al. Predicting adverse events in children with fever and chemotherapy-induced neutropenia: the prospective multicenter SPOG 2003 FN study. J Clin Oncol 2010;28:2008-14.

9. Rondinelli PI, Ribeiro Kde C, de Camargo B. A proposed score for predicting severe infection complications in children with chemotherapy-induced febrile neutropenia. J Pediatr Hematol Oncol 2006;28:665-70.

10. Santolaya ME, Alvarez AM, Becker A, et al. Prospective, multicenter evaluation of risk factors associated with invasive bacterial infection in children with cancer, neutropenia, and fever. J Clin Oncol 2001;19:3415-21.

11. Badiei Z, Khalesi M, Alami MH, et al. Risk factors associated with life-threatening infections in children with febrile neutropenia: a data mining approach. J Pediatr Hematol Oncol 2011;33:e9-e12.

12. Hakim H, Flynn PM, Srivastava DK, et al. Risk prediction in pediatric cancer patients with fever and neutropenia. Pediatr Infect Dis J 2010;29:53-9.

13. Agyeman P, Aebi C, Hirt A, et al. Predicting bacteremia in children with cancer and fever in chemotherapy-induced neutropenia: results of the prospective multicenter SPOG 2003 FN study. Pediatr Infect Dis J 2011;30:e114-9.

14. Santolaya ME. [Febrile neutropenia in the child with cancer. Current concepts about risk assessment and selective management]. Rev Med Chil 2001;129:1449-54.

15. Rackoff WR, Gonin R, Robinson C, Kreissman SG, Breitfeld PB. Predicting the risk of bacteremia in childen with fever and neutropenia. J Clin Oncol 1996;14:919-24.

Appendix:

Amendments after start of analysis

Changes from Plan of analysis version 1.0 of February 11, 2021, to version 1.1 of June 6, 2021

1) Primary analysis:

Change of the time span used for primary analysis from "reaching 38.5°C to start of antibiotics" to "reaching the randomized fever limit (38.5°C or 39.0°C) to start of antibiotics".

2) Propensity score:

Change in creation of propensity scores:

Initially it was planned to only include variables significantly associated with TTA in unavailable analysis into the multivariable models. Instead of this, the multivariable models for the first score now includes all variables that are clinically evident or known to influence the risk for SRE. The second score now includes all variables available and known at presentation. Construction of the third propensity score did not change, nor did the classification of variables to the restricted or inclusive score.

Additionally location at FN diagnosis was added to all three models, whether significant or not, to adjust for the study design.

3) Exclusion of episodes

No exclusion of episodes with early arrival and waiting to cross the current fever limit at the hospital (n=67) or starting antibiotics with delay without reaching the current fever limit (n=11) from the time "reaching the current fever limit (38.5°C or 39.0°C) to start of antibiotics".
